# Omega-3 Eicosapentaenoic Acid Is Related to Happiness and a Sense of Fulfillment—A Study among Female Nursing Workers

**DOI:** 10.3390/nu12113462

**Published:** 2020-11-11

**Authors:** Hirohito Tsuboi, Hiroyuki Sakakibara, Masahiro Matsunaga, Asami Tatsumi, Kimiko Yamakawa-Kobayashi, Naoko Yoshida, Kayoko Shimoi

**Affiliations:** 1 Institute of Medical, Pharmaceutical & Health Sciences, Kanazawa University, Kanazawa 920-1192, Japan; naoko@p.kanazawa-u.ac.jp; 2 Department of Neurology and Internal Psychosomatic Medicine, Bantane Hospital, Fujita Health University School of Medicine, Nagoya 454-8509, Japan; matsunag@aichi-med-u.ac.jp; 3 Faculty of Agriculture, University of Miyazaki, Miyazaki 889-2192, Japan; hiroyuki@cc.miyazaki-u.ac.jp; 4 School of Food and Nutritional Sciences, University of Shizuoka, Shizuoka 422-8526, Japan; kobayasi@u-shizuoka-ken.ac.jp (K.Y.-K.); shimoi@u-shizuoka-ken.ac.jp (K.S.); 5 Department of Health and Psychosocial Medicine, School of Medicine, Aichi Medical University, Nagakute 480-1195, Japan; 6 Department of Nursing, University of Human Environments, Obu 474-0035, Japan; a-tatsumi@uhe.ac.jp

**Keywords:** docosahexaenoic acid, eicosapentaenoic acid, α-linolenic acid, happiness, linoleic acid, a sense of fulfillment

## Abstract

*Background:* Omega (ω) 3 fatty acid (FA) is a polyunsaturated FA (PUFA) that can modulate some mental statuses. However, most studies have not considered the functional differences between eicosapentaenoic acid (EPA) and docosahexaenoic acid (DHA). We investigated associations among happiness, a sense of fulfillment and serum ω3 PUFA levels. *Methods:* Participants were 133 female staff from a hospital and nursing homes. Happiness was measured using the Japanese version of the subjective happiness scale (SHS); a sense of fulfillment was assessed using a visual analogue scale. Serum FA concentrations were measured. A partial correlation test and a regression model were applied. *Results:* The SHS scores showed significantly positive correlations with a sense of fulfillment, DHA% and EPA% (*p* < 0.05, < 0.05 and < 0.005, respectively), after controlling for age, BMI, menopause, snacking habits and leisure-time physical activities. A sense of fulfillment was significantly negatively correlated with α-linoleic acid%, and positively correlated with DHA% and EPA% (*p* < 0.05, < 0.05 and < 0.005, respectively), after controlling for the confounders. A regression model showed that a sense of fulfillment, EPA, and not stopping menstruation explained happiness (standardised beta, *B* = 0.18, *p* < 0.05; *B* = 0.24, *p* < 0.01; and *B* = 0.32, and *p* < 0.05, respectively), whereas age, BMI and snacking habits could not. Simultaneously, a regression model could not explain the association between DHA and happiness. *Conclusion:* Happiness was related with serum EPA%, a sense of fulfillment, and premenopause.

## 1. Introduction

Omega (ω)3 fatty acid (FA) is a polyunsaturated FA (PUFA) that has been shown to modulate depression and some other mental states [1,2,3]. However, most studies have not considered functional differences between eicosapentaenoic acid (EPA) and docosahexaenoic acid (DHA), two well-known ω3 PUFAs. These two ω3 PUFAs are often referred to generally as ω3 or *n*-3 FA, without any further distinction, and most studies have been conducted using mixtures of EPA and DHA.

The concentrations of EPA in the tissues of the central nervous system (CNS) are far lower than those of DHA [4]. DHA is particularly abundant in the human brain, and DHA and EPA enter the brain at similar rates [4]. However, EPA can be highly neuroactive [5] and may be more effective than DHA for the treatment of psychiatric disorders [3]. Some studies have shown different functions and differences in metabolism between EPA and DHA. For example, a study performed in rats showed that EPA-supplemented diets could enhance EPA levels but not DHA levels in the brain, and prevent interleukin (IL)-1β-induced inflammation [6]. Furthermore, EPA supplementation upregulated the expression of brain-derived neurotrophic factor (BDNF) in the brain [6]. Other studies have demonstrated that EPA was more effective than DHA for improving depression-like behaviours induced by chronic, unpredictable, mild stress in rats, and EPA was reported to be more effective than DHA for the inhibition of neuroinflammation, the promotion of neurotrophic factors such as BDNF, and the protection against cell death in the hippocampus [7]. However, the mechanisms underlying the differences between EPA and DHA are not fully understood.

The function of α-linolenic acid (ALA), an ω3 essential PUFA, is not fully understood. A longitudinal study involving women showed that ALA was significantly associated with a lower risk of clinical depression, especially among those who reported the lowest intake of linoleic acid (LA) [8]. ALA can be metabolically converted into EPA and DHA, the conversion of which is notably greater in women than in men [9]. However, a diet high in LA reduces the conversion of ALA into its long-chain derivatives (EPA and DHA) [10]. Although DHA and arachidonic acid (AA) are abundant in the brain compared with the levels of other PUFAs [4], small quantities of other PUFAs may be also play important roles in the mediation of brain function. Generally, ω3 and ω6 PUFAs are thought to work dynamically in the brain [8,11].

A systematic review written from the perspective of positive psychology revealed that positive emotions are directly related to improvements in depression levels [12]. A study showed that the presence of an emotional disorder and higher levels of symptom severity can affect happiness and emotional wellbeing [12]. Feelings of happiness can partly depend on eudaimonic factors, such as feeling worthwhile, having a sense of fulfillment or a psychological reward [13]. Determining methods to increase positive feelings, such as happiness, can be significant for maintaining psychological wellbeing and treating mental disorders [12], although happiness and the treatment of mental disorders, such as depression, may not exist on the same conceptual axis, because emotions are associated with multi- mediators [14].

We have been investigating factors that can help people to maintain a positive psychosomatic status in everyday life. Food and nutrition are significant factors in people’s everyday lifestyle. The intake of ω3 PUFA can provide a protective function against cognitive or psychiatric disorders [15] and may help maintain a positive psychological status. For instance, ω3 PUFA can positively affect psychological conditions [2], the effects of which have often been observed in females [15]. In addition to eudaimonic wellbeing, such as worthwhile feelings [16], nutritional factors can also help to maintain hedonic wellbeing, such as feelings of happiness. In the present study, therefore, we investigated associations among subjective happiness and a sense of fulfillment, namely feelings of being worthwhile, and serum concentrations of PUFA with confounders.

The objective of this study was to examine the associations among a sense of fulfillment, happiness and ω3 PUFA levels in serum and to investigate the functional differences between EPA and DHA.

## 2. Materials and Methods

### 2.1. About This Study

Data from this study have previously been published elsewhere, showing interrelationships among serum ω3 PUFA levels, subjective stress and depressive symptoms [17]. We did not publish any results concerning the association between ω3 PUFA and positive feelings because in 2009, when the study was performed, it was hard to explain the mechanism of any relationship between them. However, recent studies on the functions of PUFA have made it possible to explain the relationship. 

The Ethics Committee of the Fujita Health University School of Medicine in Aichi, Japan, approved the study protocol (Protocol #09-098), and all participants gave written informed consent before enrollment.

### 2.2. Participants and Procedures

A cross-sectional study was performed involving 140 female nursing and nursing care workers recruited from a hospital and two nursing homes in the Shizuoka Prefecture of Japan. No participants had been diagnosed with any psychotic disorders, and all of them worked regularly. The participants’ mean age was 45.7 years (standard deviation (SD): 13.0; range: 19–75 years). 

Data were collected at the time of the participants’ annual health check-up, which was performed over the course of four days in August 2009. A self-administered questionnaire was distributed to the participants beforehand and was completed one day prior to examination. The participants’ privacy was protected during the entire process. 

### 2.3. Questionnaire

The questionnaire contained questions regarding demographic measures (age, present health status, medication, menstruation, etc.); lifestyle characteristics, such as smoking status (non-smoker or ex-smoker vs. current smoker); alcohol consumption (almost never vs. ≥once per week); eating breakfast (everyday vs. sometimes or never); snacking habits (eat often vs. not so often); leisure-time physical activities (once per month vs. ≥once per week); and psychological measures. We assessed the participants’ subjective happiness levels using the Japanese version of the Subjective Happiness Scale (SHS) [18,19]. In this survey, the scale was determined to present sufficient internal consistency, with a Cronbach’s α value of 0.80. A sense of fulfillment was also evaluated in the questionnaire, using a visual analogue scale (VAS), which comprised a horizontal, non-calibrated line, 10 cm in length, ranging from ‘not at all’ (0) to ‘quite strong’ (100) [20]. Participants were asked to answer questions, such as “Do you have anything rewarding or fulfilling in your daily life like a hobby, job, etc.?” 

### 2.4. Assays

#### 2.4.1. Blood Collection

Blood was collected in serum-separator vacutainer tubes from each participant’s forearm vein, between 08:30 and 10:30 am and after they had fasted for 12–14 h; the sera were isolated by centrifugation. Serum samples were shipped to a commercial laboratory (FALCO Biosystems Ltd., Kyoto, Japan), where measurements were performed within 3 weeks.

#### 2.4.2. Lipid and PUFA Assays

Serum triglycerides (TG), total cholesterol (TC), and non-esterified fatty acids (NEFA) were measured enzymatically. A gas chromatographic method was used to determine FA levels (FALCO laboratory). The following types of FAs were analyzed: ω6 PUFAs (LA, AA, dihomo-γ-linolenic acid, γ-linolenic acid, eicosadienoic acid, and docosatetraenoic acid) and ω3 PUFAs (DHA, EPA, ALA and docosapentaenoic acid).

#### 2.4.3. Statistical Analysis

Prior to data analysis, six participants with serum TG levels of more than 2.48 mMol/L were excluded, because chylous serum, caused by high TG levels, may hinder the analysis. Consequently, 133 participants were included in the analysis.

The Japanese version of SPSS Statistics 25 was used (IBM, Tokyo, Japan) to perform the data analysis. For comparisons of two values, a linear model was used, for controlling for possible confounders, following *t*-test. To assess correlations between two values, partial correlations were used to adjust for covariates that might affect the results, followed by simple correlation tests. Multiple linear regression analyses were applied to detect factors that were significantly associated with SHS scores. Through discussions, we developed a mediation analysis, using SPSS Process Macro version 3.5 [21], to estimate how PUFA mediates between a sense of fulfillment and happiness. Significance was reported at *p* < 0.05.

## 3. Results

### 3.1. Participant Characteristics and PUFA

Table 1 presents the demographic data, psychological variables, lifestyle parameters, and serum lipid and PUFA concentrations for participants included in the analysis.

Serum PUFA concentrations were strongly positively correlated with each other and with NEFA, TC and TG levels; this is because NEFA, TC and TG contain and transport FA in the serum. Thus, PUFA levels should be adjusted to not be affected by each other for analyzing the functions. We adjusted the level of each ω3 and ω6 PUFA by dividing total FA concentrations. In addition, because menopause depends on age, and because PUFA distribution varies according to body mass index (BMI), differences in PUFA ratios in physical, lifestyle and social factors were compared while controlling for age and BMI.

Table S1 shows the differences in PUFA% in relation to physical, social and lifestyle factors. DHA% and ALA% were significantly higher in menopaused participants compared with their levels in participants with menstruation after controlling for age and BMI (*p* = 0.002 and 0.012, respectively). Concerning snacking habits, ALA% was significantly higher in participants who never or seldom ate between meals than those who snacked regularly (*p* = 0.034). Participants who exercised one or more times per week showed significantly higher AA% than those who exercised less than a few times per month (*p* = 0.017). There were no significant differences between PUFA% with the habit of eating breakfast, smoking status, alcohol consumption or occupational status.

### 3.2. Correlation between Psychological Factors an Levels of Serum PUFA

Table 2 presents partial correlations among SHS scores, a sense of fulfillment and PUFA%, controlling for age, BMI, menopause, snacking habits and leisure-time physical activities.

**Figure 1:**
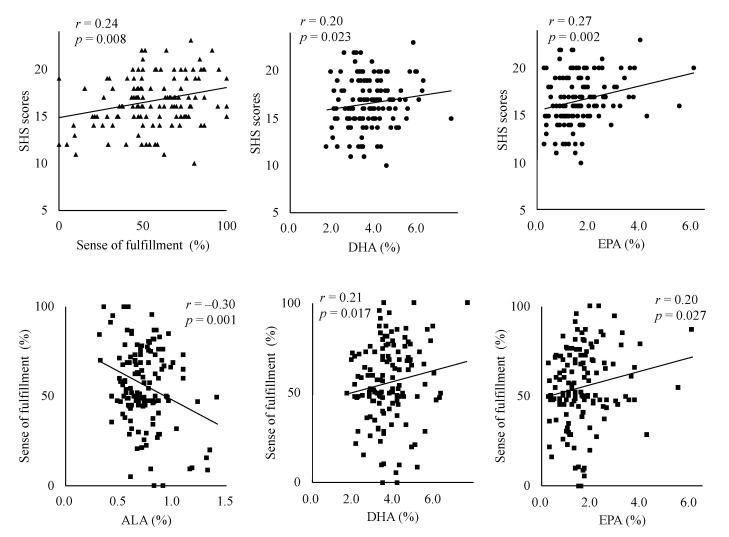
Significant partial associations between happiness as assessed by SHS, a sense of fulfillment, and fatty acid levels, adjusted for age, BMI, menopause, snacking habits, and leisure-time physical activities. The SHS scores showed significant positive correlations with a sense of fulfillment, DHA, and EPA percentage. (*p* < 0.05, < 0.05 and < 0.005, respectively). A sense of fulfillment showed significant negative correlations with ALA percentage, and significant positive correlations with the percentages of DHA and EPA (*p* < 0.005, < 0.005 and < 0.05, respectively). SHS: subjective happiness scale, LA: linoleic acid, EDA: eicosadienoic acid, ALA: α-linolenic acid, DHA: docosahexaenoic acid, EPA: eicosapentaenoic acid.

The SHS scores were significantly positively correlated with fulfillment, DHA% and EPA% (*p* = 0.008, *p* = 0.023 and *p* = 0.002, respectively). A sense of fulfillment showed significantly positive correlations with the concentrations of DHA% and EPA% (*p* = 0.017 and 0.27, respectively), whereas a sense of fulfillment showed a significant negative correlation with ALA (*p* = 0.001). These significant correlations are shown in Figure 1.

### 3.3. Regression Models to Explain Happiness

Table 3 shows regression models that were used explain happiness. All models explained that a sense of fulfillment was positively significantly related with SHS scores (*p* < 0.05). In Model 1, menopaused participants were found to be significantly less happy compared with those with menstruation (*p* < 0.05), whereas DHA% showed no significant relation with SHS scores. In Model 2, SHS scores were significantly positively related with EPA% and having menstruation (*p* < 0.05 and < 0.01, respectively). In Model 1+2, the significant associations between happiness, EPA and menopause disappeared. However, this model explains 9% of happiness (adjusted *R*^2^ = 0.09), whereas Model 2 for EPA explains 10% of happiness (adjusted *R*^2^ = 0.10).

## 4. Discussion

Associations among happiness, a sense of fulfillment (feelings of being worthwhile), and serum PUFA% in females working at a hospital and two nursing homes were explored. Our regression model showed that happiness could be significantly explained by higher serum EPA%, a higher sense of fulfillment and premenopause. In contrast, the regression model could not explain happiness by DHA%.

Partial correlations revealed significant positive correlations between happiness with DHA%, EPA%, and a sense of fulfillment. A sense of fulfilment was significantly positively correlated with DHA% and EPA%, whereas it was significantly inversely correlated with ALA%. The correlation coefficient of EPA was higher than that of DHA.

### 4.1. ALA and Emotion

The inverse relationship between ALA and a sense of fulfillment identified in the present study may be due to the conversion of ALA into DHA and EPA, and a sense of fulfillment might stimulate this conversion of ALA. Although the function of ALA is poorly understood, some studies have reported the in vivo kinetics of ALA and the existence of a relationship between ALA and depression. Large amounts of dietary ALA are rapidly catabolised into carbon dioxide, resulting energy, and ALA has the highest rate of oxidation among all unsaturated FAs [10]. The inverse relationship between ALA and a sense of fulfillment suggested that eudaimonic wellbeing, resulting from an increase in a sense of fulfillment, may have increased ALA oxidation, because psychological stress can cause oxidative stress, regardless of whether it derives from eustress or distress [22]. In addition, catabolysis to carbon dioxide to liberate energy represents a type of oxidative eustress that occurs as a biological reaction and is not associated with negative effects on the body or brain [23]. Although one study indicated an association between an increased dietary intake of ALA and a reduced risk of depression [8], dietary intake volumes are not necessarily reflected in the peripheral blood concentration, and that depression is a kind of results while a sense of fulfillment is not. Thus, we inferred that large quantities of ALA were catabolised and that small amounts of ALA were converted into EPA.

### 4.2. EPA and Happiness

EPA showed a positive correlation with SHS scores. EPA and DHA appeared to be associated with happiness (Table 2); however, the association between DHA and happiness disappeared in the regression model (Table 3). EPA and DHA may differ in their mechanism of action on brain function. Although these two well-known ω3 PUFAs have often been studied in combination, they have rarely been examined separately. 

DHA and AA are abundant in the human brain [10]; however, the concentration of EPA in the CNS is much lower than that of DHA, even though the fact that EPA and DHA enter the brain at a similar rate [24]. DHA is rapidly incorporated into phospholipids in the brain, maintaining it at high levels [25], whereas EPA is slowly incorporated and extensively metabolized [24]. These data suggest that EPA may primarily function outside of the CNS, unlike DHA.

Pure EPA supplementation improved impaired endothelium-dependent blood vessel relaxation [26]. A community-based prospective cohort study, performed in older adults with heart failure, indicated the existence of an inverse association between incidence of coronary heart failure and EPA concentrations among plasma phospholipids; conversely, no association was found with DHA [27]. Some studies using molecular indicators also indicated that EPA plays a role in the endothelium. A cohort study performed in adults with atherosclerosis reported that high plasma levels of EPA and EPA + DHA were associated with lower levels of soluble forms of intercellular cell adhesion molecule (ICAM)-1 in participants who were obese [28]. Moreover, a meta-analysis revealed that EPA supplementation decreased the circulating levels of adhesion molecules, including soluble ICAM-1 and soluble forms of vascular cell adhesion molecule (sVCAM)-1, both in participants with dyslipidemia and in healthy controls [29]. ICAM-1 and VCAM-1 are examples of inflammatory adhesion molecules that contribute to vascular endothelial functional properties. Another meta-analysis reported that the antidepressant activity of EPA is superior to that of DHA, with pure DHA failing to reduce depression [30]. Although many studies have examined depression and ω3 PUFAs, studies examining the association between ω3 PUFAs and happiness are sparse. However, studies concerning depression are beneficial for discussions of happiness because endocannabinoids (eCBs) may also act on the remission of depression [31,32]. eCBs are an important class of biologically active FA-derivatives, which have been identified as cannabinoids or a cannabis-like compound [10]. eCB is present and is active in the human body, even if one has not ingested cannabis. Endogenous 2-arachidonylglycerol (2-AG), one of the main eCBs, contributes to the restoration of inflammation-related microvasculature dysfunction caused by overoxidation [33]. Endogenous 2-AG abolished VCAM-1 and ICAM-1 elevation induced by CB1/2 receptor activation [33]. The pleiotropic effects of endogenous 2-AG may prevent and treat depression by abolishing inflammation and vasculature dysfunction. The clinical remission of depression has been correlated with plasma levels of eicosapentaenoyl ethanol-amide (EPEA), which is an EPA-derived eCB metabolite [1]; EPA has an affinity for CB1/2 receptors that is 100- to 1000-fold higher than those of DHA derivatives [34]. Taken together, these findings suggest that EPA might decrease depressive symptoms and evoke happiness via endothelial anti-inflammatory reactions. 

### 4.3. Speculation about the Mediation between a Sense of Fulfillment and Happiness

We developed a mediation model to elucidate a pathway from a sense of fulfillment to happiness. Although it is inappropriate to suggest causal relationships based on the present cross-sectional study, it is possible to speculate on the pathway according to a psychological relationship between cause and effect [16]. Mediation analysis tests whether a relationship between two variables can be explained by a third intermediate variable [35]. This has mainly been used mainly used in psychological research.

**Figure 2:**
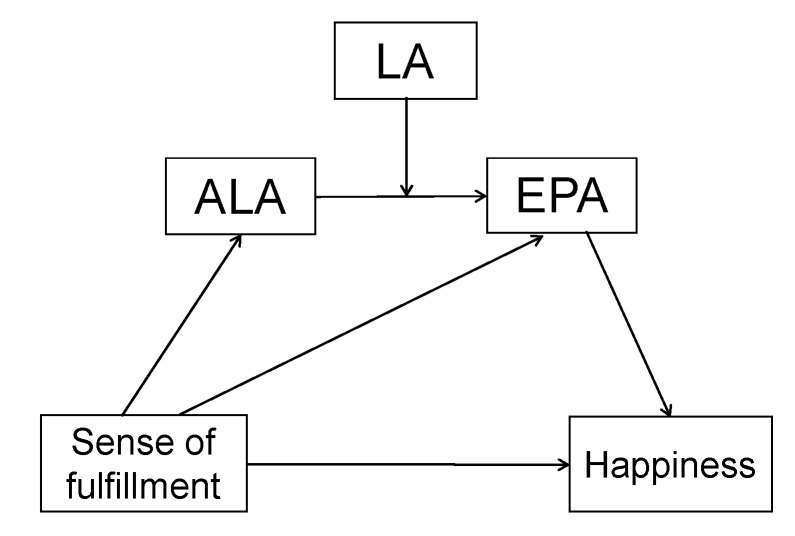
A mediation model to explore the underlying processes between a sense of fulfillment and happiness. This figure presents a hypothetical model based on existing research and statistical analysis: ALA oxidation by eustress, and an LA function that affects the conversion of ALA into EPA. LA did not show a significant effect on the conversion of ALA to EPA. No indirect effects from ‘a sense of fulfillment to SHS’ were significant. Only a direct effect of ‘a sense of fulfillment on SHS’ was significant (*p* < 0.005) (age, BMI, menopause, snacking habits and leisure-time physical activities were adjusted as covariates).

Figure 2 shows a hypothetical mediation model, generated using the mediation model 91 [36]. We developed this mediation model because ALA and a sense of fulfillment were inversely related with each other, because EPA was positively correlated with a sense of fulfillment and happiness, and because ALA can be converted into EPA in the presence of LA [37]. Age, BMI, menopause, and snacking habits were adjusted as covariates in the analysis.

LA did not have a significant effect on the conversion of ALA to EPA (coefficient = 0.03, *p* = 0.73). An indirect effect of ‘a sense of fulfillment → ALA → EPA → SHS scores’ was not significant (effect = −0.000, boot confidence interval (CI): −0.002 to 0.002; effect = −0.003, CI: −0.001 to 0.002; Effect = −0.001, CI: −0.001 to −0.003, respectively). A direct effect of ‘a sense of fulfillment on SHS’ was significant (effect = 0.025, p = 0.035, CI: 0.002 to 0.047). No significant mediations of ‘a sense of fulfillment → ALA → SHS’ (effect = 0.001, boot CI: −0.007 to 0.003) or ‘a sense of fulfillment → EPA → SHS’ (effect = 0.005, boot CI: −0.002 to 0.013) were identified. Consequently, this model failed to explain any indirect pathways between a sense of fulfillment and happiness. 

This was a speculative model that was generated based on the metabolic pathways of FAs and the present research results. Unfortunately, this model could not explain the indirect effects of FAs between a sense of fulfillment and happiness. This result might be due to the small sample size, or there may be other indirect factors; otherwise, however, the model may be inappropriate.

### 4.4. Limitations

The present study has some limitations that should be acknowledged. Due to the inherent nature of a cross-sectional study, causal relationships could not be verified. Future longitudinal or intervention-based studies will be necessary to address this issue. Biomarkers that may explain EPA functions, such as eCBs, EPEA, ICAM-1 and VCAM-1, were not measured. In addition, the participants comprised just 133 women, working in a hospital or in nursing homes. Participants with other occupations or of different social status, as well as male participants, should be investigated in the future.

## 5. Conclusions

We found that happiness had stronger positive relationships with a sense of fulfillment, serum EPA%, and premenopause, in that order, among a hospital and nursing home workers. Furthermore, we tried to speculate medications from a sense of fulfillment to happiness. Although our model failed to explain indirect pathways through FAs, the question remains as to why ALA and a sense of fulfillment were inversely related. To the best of our knowledge, this is the first report showing an association between happiness and EPA separate from DHA. Although additional studies, using other populations, larger sample sizes, and preferably longitudinal designs, remain necessary to further evaluate the functions of EPA, our findings suggest that EPA may be beneficial to maintain our psychological wellbeing.

## Figures and Tables

**Table 1:** Characteristics and clinical variables of the participants (*n* = 133).

Variable	Mean ± SD	Range
Age (years)	45.4 ± 13.24	19–75
BMI (kg/m^2^)	21.7 ± 3.35	15.8–32.8
SHS scores *	16.7 ± 2.82	10–28
Sense of fulfillment (%)	55.6 ± 22.00	0–100
Occupational status	*n*	(%)
Licensed nurse or caregiver	90	67.7
Others without license	43	32.3
Physical and lifestyle variables	*n*	(%)
Menopause		
No	71	53.4
Yes	62	46.6
Smoking status		
Ex- or non-smoker	103	77.4
Current smoker	30	22.6
Current alcohol consumption		
(Almost) Never	112	84.2
≥Twice per week	21	15.8
Eating Breakfast		
No or sometimes	16	12.0
Yes	117	88.0
Eating between meals		
No or seldom	57	42.9
Yes	76	57.1
Leisure-time physical activities		
<A few per month	104	78.2
≥once per week	29	21.8
Serum profile of lipids	Mean ± SD	Range
Triglycerides (mMol/L)	0.984 ± 0.4784	0.33–2.38
Total cholesterol (mMol/L)	5.51 ± 1.128	3.39–8.28
Non-esterified fatty acid (mEq/L)	0.65 ± 0.239	0.20–1.27
FA concentrations (µmol/L)	Mean ± SD	Range
ω6 PUFA	4165 ± 754.9	2763–6937
Linoleic acid (LA) *	3392 ± 623.0	2068–4806
Arachidonic acid (AA)	570.71 ± 125.79	296–1003
Dihomo-γ-linolenic acid	115.1 ± 37.87	40.5–204.2
γ-Linolenic acid *	32.77± 19.734	3.6–94.1
Eicosadienoic acid	18.46 ± 4.320	9.7–29.8
Docosatetraenoic acid	13.59 ± 4.187	0.0–24.1
ω3 PUFA	741.8 ± 296.6	265–1836
Docosahexaenoic acid (DHA)	414.7 ± 150.50	152–889
Eicosapentaenoic acid (EPA) *	184.5 ± 113.12	25.0–549.0
α-Linolenic acid (ALA)	81.77 ± 30.809	28.0–183.2
Docosapentaenoic acid	56.39 ± 21.077	19.4–121.9

BMI: body mass index. SHS: subjective happiness scale. FA: fatty acid. PUFA: poly-unsaturated FA, SD: standard deviation. * An outlier (>mean + 4SD) was excluded (*n* = 132).

**Table 2:** Partial correlations between psychological indices and significant serum PUFA levels.

Correlation Coefficient	SHS Score	Fulfillment	LA (%)	AA (%)	DGLA (%)	LNA (%)	EDA (%)	DTA (%)	DHA (%)	EPA (%) ^a^	ALA (%)	DPA (%)
SHS score												
Fulfillment	0.24 *											
LA (%)	−0.17	−0.07										
AA (%)	0.02	0.12	−0.11									
DGLA (%)	0.01	−0.04	−0.25 **	0.13								
LNA (%)	0.01	0.03	−0.45 ***	0.27 **	0.56 ***							
EDA (%)	−0.1	−0.25	0.05	−0.16	0.40 ***	−0.08						
DTA (%)	−0.03	0.01	−0.16	0.32 ***	0.54 ***	0.50 ***	0.25 *					
DHA (%)	0.20 *	0.21 *	−0.35 ***	0.09	−0.19 *	−0.17	−0.06	−0.30 **				
EPA (%) ^a^	0.27 **	0.20 *	−0.32 ***	0.095	−0.28 **	−0.09	−0.28 **	−0.35 ***	0.78 ***			
ALA (%)	−0.01	−0.30 **	−0.05	−0.44 ***	−0.06	−0.07	0.40 ***	−0.14	0.01	−0.06		
DPA (%)	0.14	0.15	−0.50 ***	0.03	−0.03	0.09	0.04	−0.09	0.81 ***	0.66 ***	0.10	

SHS: subjective happiness scale, Fulfillment: a sense of fulfillment (%), LA: linoleic acid, AA: arachidonic acid, DGLA: dihomo-γ-linolenic acid, LNA: γ-linolenic acid, EDA: eicosadienoic acid, DTA: docosatetraenoic acid, DHA: docosahexaenoic acid, EPA: eicosapentaenoic acid, ALA: α-linolenic acid, DPA: docosapentaenoic acid, BMI: body mass index. Adjusted for age, BMI, menopause, snacking habits and leisure-time physical activities. ^a^ An outlier (> mean + 4SD) was excluded (*n* = 132). * *p* < 0.05, ** *p* <0.005, *** *p* <0.0005.

**Table 3:** Regression models used to explain happiness.

Models	Model 1		Model 2		Model 1 + 2 *	
Adjusted *R*^2^	0.08		0.10		0.10	
*f*-value	2.80 (6, 124)		3.44 (6, 124)		3.00 (7, 123)	
*p*-value	0.014		0.004		0.006	
Independent variables	*B*	*p*	*B*	*p*	*B*	*p*
Sense of fulfillment	0.20	0.023	0.19	0.036	0.19	0.035
DHA	0.13	0.187			−0.09	0.573
EPA			0.25	0.025	0.32	0.058
Age	0.23	0.09	0.19	0.154	0.18	0.193
BMI	−0.04	0.633	−0.02	0.858	−0.01	0.953
Menopause	−0.29	0.046	−0.30	0.035	−0.27	0.064

*B*: Standardised beta. Dependent variable: Subjective happiness scale. * DHA × EPA interaction was included in the model.

## Supplementary Materials

The following are available online at https://www.mdpi.com/2072-6643/12/11/3462/s1, Table S1: Differences of PUFA% by physical, social and lifestyle factors.
